# Oral health survey and oral health questionnaire for high school students in Tibet, China

**DOI:** 10.1186/1746-160X-10-17

**Published:** 2014-05-19

**Authors:** Rui Hou, Yong Mi, Quanhong Xu, Fang Wu, Yuanyuan Ma, Peng Xue, Gao Xiao, Yan Zhang, Yinhua Wei, Wenbing Yang

**Affiliations:** 1 Department of Dentistry, 41st PLA Hospital, Naidong Road 80, Naidong County, Shannan prefecture, Tibet 856000, China; 2 State Key Laboratory of Military Stomatology, Department of Oral and Maxillofacial Surgery, School of Stomatology, the Fourth Military Medical University, Changle West Road 145, Xi’an, Shaanxi 710032, China

**Keywords:** Oral health, Questionnaire, Epidemiology, Tibet, Plateau

## Abstract

**Objectives:**

The aim of this study is to identify the oral health status as well as oral health practices and access for care of graduating senior high school Tibetan students in Shannan prefecture of Tibet.

**Methods:**

Based on standards of the 3rd Chinese National Oral Epidemiological Survey and WHO Oral Health Surveys, 1907 graduating students from three senior high schools were examined for caries, periodontitis, dental fluorosis, and oral hygiene status. The questionnaire to the students addressed oral health practices and present access to oral medical services.

**Results:**

Dental caries prevalence (39.96%) and mean DMFT (0.97) were high in Tibetan students. In community periodontal indexes, the detection rate of gingivitis and dental calculus were 59.50% and 62.64%, respectively. Oral hygiene index-simplified was 0.69, with 0.36 and 0.33 in debris index-simplified and calculus index-simplified, respectively. Community dental fluorosis index was 0.29, with 8.13% in prevalence rate. The questionnaire showed students had poor oral health practices and unawareness for their needs for oral health services. It was also noted that the local area provides inadequate oral medical services.

**Conclusions:**

Tibetan students had higher prevalence of dental diseases and lower awareness of oral health needs. The main reasons were geographical environment, dietary habit, students’ attitude to oral health, and lack of oral health promotion and education. Oral health education and local dentists training should be strengthened to get effective prevention of dental diseases.

## Introduction

Tibet lies on the Qinghai-Tibet Plateau of the southwest border of China. The average elevation of the whole region is over 4,000 meters above sea level, and commonly referred to the “Roof of the World”. The region covers more than 1.2 million square kilometers, accounting for one eighth of Chinese total land mass, and ranking second in China.

The complex terrain and diverse topography of the plateau provide a unique climate. It has rarefied air and low atmospheric pressure, with oxygen content only two third of that at sea level. It has strong solar radiation and long hours of sunlight exposure with a marked difference in temperature between day and night. The whole year can be divided into two distinct dry and wet seasons. In winter and spring, there is little rain and frequent strong winds, with lower oxygen content in the atmosphere.

Although a part of China, Tibet has a unique culture of its own. Of all the residents in the region, ethnic Tibetans number 2.41 million, accounting for 92.2 percent of the total Tibet population.

The Tibetans have their unique customs, living environment, and dietary habit which subject them to distinct dental disease characteristics.

Although nationwide oral epidemiology investigations were done in 1983, 1995, and 2005 in China, Tibet was not included and there are only a few reports on Tibetan oral health status, and most of them were done without adhering to appropriate standards [1-3].

In this study, we conducted an oral health examination based on standards of the 3rd Chinese National Oral Epidemiological Survey [4] and WHO Oral Health Surveys [5]. The Tibetan students were examined for caries, periodontitis, dental fluorosis, and oral hygiene status. The students’ questionnaire addressed oral health practices, present access to oral medical services, and the anticipated needs of oral health services. The related reasons for the oral health status were also analyzed.

## Materials and methods

The research had the approval of the 41st PLA hospital’s Ethics Committee (No.2013-04-02), which was in compliance with the Helsinki Declaration.

Graduating students from all three senior high schools in Shannan prefecture had an oral examination by specialists who had already been trained for the same standards before the 2013 College Entrance Examination. The standards of the examination were based on the 3rd Chinese National Oral Epidemiological Survey and WHO Oral Health Surveys. The specialized oral health examination was performed in accordance with World Health Organization (WHO) diagnostic criteria, for caries, periodontitis, dental fluorosis, and oral hygiene status. The results for each item are presented separately and listed below. (1) Caries index (including DMFT Decayed Missing Filled Teeth, DMFS Decayed Missing Filled Surfaces, Mean DMFT, caries prevalence rate). (2) Community periodontal index (including detection rate of gingivitis and dental calculus). (3) Oral hygiene index-simplified (including debris index-simplified and calculus index-simplified). (4) Community dental fluorosis index (including prevalence rate of dental fluorosis). Questionnaire Students’ age, gender, place of residence (urban or rural areas) were collected from all students. The students were asked about their oral health practices, present access to oral medical service, and their current needs of oral health services.

Written informed consent was obtained from the student for the publication of this report and any accompanying images.

### Statistical analysis

SPSS version 13.0 for Windows was used for statistical analysis. The descriptive statistics were made on a different index. Independent sample test was used to compare the scores on various indexes between gender and the place of residence of students.

## Results

A total of 1907 students, 858 male and 1049 female, with a mean age of 18.93 years (range 16–23), received the oral examination and completed the questionnaire. Of all the students, 1324 came from rural areas and 583 from urban areas.

### Oral health examination

The results indicated [1] DMFT was 1845 and DMFS was 7184. Of all the DMFT, the percent of decayed, missing, and filled teeth were 88.36%, 9.81%, 1.83%, respectively. Mean DMFT was 0.97, with caries prevalence rate of 39.96%. Caries prevalence rate of first molars was up to 32.25%. [2] In community periodontal indexes, the detection rate of gingivitis and dental calculus were 59.50% and 62.64%, respectively. [3] Oral hygiene index-simplified was 0.69, and debris index-simplified and calculus index-simplified were 0.36 and 0.33, respectively. [4] Community dental fluorosis index was 0.29, with prevalence rate of 8.13%. Table 1 shows the calculated indexes of different genders and place of residence for the students. The significant difference was between students from rural areas and those from urban areas.

**Table 1:** The oral indexes of different gender and source of students

	**Male**	**Female**	**Students from rural areas**	**Students from urban areas**	**Average**
Mean DMFT	0.79	1.12	0.39	1.48*	0.97
Caries prevalence rate (%)	34.49	44.42	16.16	64.00*	39.96
Detection rate of gingivitis (%)	61.90	58.72	63. 24	56. 13	59.50
Detection rate of dental calculus (%)	63.34	61.02	64. 22	60. 51	62.64
OHI-S	0.78	0.63	0.70	0.66	0.69
DI-S	0.41	0.33	0.35	0.38	0.36
CI-S	0.37	0.30	0.35	0.28	0.33
CFI	0.23	0.35	0.42	0.15*	0.29
Prevalence rate of dental fluorosis (%)	6.18	9.72	11.86	5.34*	8.13

*P < 0.05, the significant difference was between students from rural areas and those from urban areas.

### Questionnaire

#### Oral health practice

Table 2 shows results of oral health practices of students. The number of students who brushed teeth once a day (72.89%) was much higher than those who brushed teeth no more than once and twice. There were also more students accustomed to use herbal toothpaste (58.57%) than other toothpaste. 74.41% of the students did not have the habit of gargling after dinner.

**Table 2:** Oral health practice of students [number (%)]

	**0**	**1**	**2**	**3**
Toothbrushing frequency every day	150(7.87%)	1390(72.89%)	367(19.24%)	0(0%)
Toothpaste daily used	639(33.51%)	1117(58.57%)	148(7.76%)	3(0.16%)
Gargling habits after dinner	1419(74.41%)*	488(25.59%)	--	--

Note:

Toothbrushing frequency every day: 0 = no more than once, 1 = once, 2 = twice, 3 = more than twice.

Toothpaste accustomed to using: 0 = ordinary toothpaste, 1 = herbal toothpaste, 2 = fluoride toothpaste, 3 = unclear.

Gargling habit after dinner: 0 = No, 1 = Yes.

### Present access to oral medical service

Table 3 shows present access to oral medical services. Of all the 1907 students, there were only 65 students who have been to the dentist in the previous year. 59 students (3.09%) had been once, 6 (0.31%) had been twice and no one had visited the dentist three times. Of the 65 students, 35 went to the dentistry department at the general hospital, while the other 30 went to the private dental clinics. No one went to a stomatological hospital. The students provided three main reasons for not consulting the dentists. The first reason was that good or bad teeth do not matter (1053 students, 55.22%). The second reason was being busy with school work and no time to visit the dentist (675 students, 35.39%). The third reason was their lack of awareness of having dental diseases (179 students, 9.39%). Of all the students, 551 students (28.89%) previously asked for sick leave because of dental diseases.

**Table 3:** Present access to oral medical service for students [number (%)]

	**1**	**2**	**3**	**4**
Times to see the dentists	59(3.09)	6(0.31)	0	1842(96.59)
Place for seeing the dentists	35(1.84)	30(1.57)	0	--
Reasons for not to see the dentists	1053(55.22)	675(35.40)	179(9.39)	--
Ask for sick leave because of dental diseases	551(28.89)	1356(71.11)	--	--

Notes:

Times to see the dentists: 1 = once, 2 = twice, 3 = three times, 4 = none.

Place for seeing the dentists: 1 = dentistry department of general hospital, 2 = private dental clinic, 3 = stomatological hospital.

Reasons for not to see the dentists: 1 = good or bad teeth do not matter, 2 = being busy with study, no time. 3 = hadn’t known they had dental diseases (179 students, 9.39%).

Asked for sick leave because of dental diseases: 1 = Yes, 2 = No.

### Need of oral health service

Of all the students, 893 (46.83%), thought they needed oral health instruction, 504 (26.43%) needed oral examination, and 119 (6.24%) needed dental diseases treatment.

## Discussion

Tibet is a plateau region in southwest of China and the home to the indigenous Tibetan people; one of the main ethnic groups in China. The area has high elevation with rarefied and oxygen-deficient air. Because of the unique plateau climate and large rugged terrain, the population density is low. The limited availability for travel and transportation caused by this distinct landscape resulted in underdeveloped industries, agriculture and farming. Because of the comparatively slow development, Tibet is considered underserved with a much lower rate of disease prevention and treatment than those in other areas of China.

According to the previous reports [1-3], the Tibetans seemed to have higher prevalence of caries, dental fluorosis and periodontitis than the majority Han ethnic group. This study showed that caries prevalence rate was 39.96%, and mean DMFT was 0.97. This was significantly higher than those of 12-year old group (caries prevalence rate was 29.0%, and mean DMFT was 0.55) reported in the results of the 3rd Chinese National Oral Epidemiological Survey [4]. There was no comparable Chinese studies that examined the same parameters for 18 year old group of people. The data was also much higher than those of other minority groups in southwest of China in the surveys of 1995 and 2005 [6]. The percent of decayed, missing, and filled teeth revealed that only about 2% of teeth got treated, including many bad fillings. Most students did not seek treatment for decayed teeth and only had some medicine to relieve pain. Further analysis found that caries prevalence rate and mean DMFT were significantly higher in students from urban areas than those from rural areas (P < 0.05). Additionally, caries prevalence rate was especial high in first molars, which suggested neglected dental health since childhood.

Although there were no national data to compare both of community periodontal index and oral hygiene index-simplified (including debris index-simplified and calculus index-simplified), the detection rate of gingivitis and dental calculus were significantly higher than those of 12-year old group (57.7% and 59.0%) in the 3rd Chinese National Oral Epidemiological Survey [4], indicating poor oral hygiene status in Tibetan students.

According to the results of community dental fluorosis index and fluorosis prevalence rate, Tibet was still demonstrating high prevalence for dental fluorosis. However, students from rural areas had significantly higher community dental fluorosis index and higher prevalence rate (P < 0.05) than students from urban areas.

The questionnaire revealed that students had poor oral health practices. It showed that more than 80% students had no habit of brushing their teeth twice a day. Nearly 60% students accustomed to use herbal toothpaste than other toothpaste. And almost three third of the students did not have the habit of gargling after dinner.

The questionnaire also showed that present situation of oral medical services was not optimistic. Although prevalence rate of caries, periodontitis, and dental fluorosis was high and up to 28.89% of the students once asked for sick leave because of dental diseases, only 3.41% of the students went to see dentists in the previous year. More than half of the students considered good or bad teeth do not matter, more than one third of students had no time to seek treatment, and nearly ten percent of students didn’t know they had dental diseases.

Given the lack of local stomatological hospital to provide advanced care, only limited oral medical services were available to the patients. As for the low awareness for the need of oral health services, Oral health instruction, oral examination, and treatment were still not considered important for the students in the survey.

According to the findings from the study, we analyzed the possible reasons listed below.

The first and most important finding was that Tibet was still demonstrating high prevalence for dental fluorosis, especially in rural area. This is mainly because the Tibetans, since childhood, have the habit of drinking tea with high content of fluorine [3,7,8]. When having traditional buttered tea, sweet tea, and zanba, a kind of roasted qingke barley flour, they always stir and mix brick tea with them. The brick tea is made of cheap, old and rough tea leaves which have higher content of fluorine than tender leaves [3,7,8]. Thus, the endemic fluorosis in Tibet, also called brick-tea-type fluorosis, was essentially due to heavy consumption of foods prepared with brick-tea. Therefore, fluoride toothpaste should not be suggested, especially for students from the rural areas.

The reason for the second finding is that eating practices in the region are constantly changing. The traditional Tibetan foods are zanba, yak and mutton with less vegetable and fruit which is particularly noted in rural areas where cultivation is low and outside food supply is short.

With the increasing exposure to the Han’s culture and custom, Tibetans have acquired similar eating habits to the rest of China. The middle school students like modern processed food, such as candies and cookies, much more than natural food. The sugary diet makes them more susceptible to the dental caries, especially in students from urban areas.

The reason for the third finding is people’s poor attitude to oral health and the lack of public oral health promotion in Tibet.

Wehmeyer MM et al. [9] found that the effect of oral health literacy (OHL) on periodontal health status remained statistically significant (P < 0.002) even after allowing for smoking, race, and dental insurance, concluding that lower OHL was associated with more severe periodontal disease among the surveyed patients. Jürgensen N et al. [10] also considered that attitude towards health were important predictors for oral health behavior and risk behavior in the Lao PDR. Likewise, the Tibetans did not seek the need to see the dentist when they had toothache or gingival bleeding since dental diseases usually do not threaten one’s life or have a bad effect on their health compared with infectious diseases, traumatic injury, or respiratory diseases.

The poor attitude towards oral health may be related to the lower educational level and reduced socio-economic condition in Tibet when compared to other parts of China. The same view was observed by Christensen LB et al. [11], who found low education level was one of the important determinants for high level of caries in children. More than 80% of people in Tibet are farmers and herdsmen with limited education and lesser amount of knowledge about general and oral health care. A report on oral health status of 200 Tibetan pupils in 2008 showed that 99% of pupil didn’t brush their teeth before going to bed and 99.5% percent of pupil practiced incorrect methods of brushing teeth [12].

Additionally, there is almost no project for public information and education about oral health in Tibet. Tibetans have no access to information on oral health provided to other Chinese through various channels. Even students could hardly get any oral health knowledge from schools because there are no teachers that could give such lessons. In reality, knowledge provided through schools could motivate individuals to adopt a healthy behavior [13]. Chandrashekar BR et al. [14] found the concept of utilizing teachers for dental health education (DHE) is practical to promote oral hygiene. And frequent DHE by teachers was more effective than the infrequent DHE by the professionals. For all those reasons, there is an urgent need for oral health education and promotion that teaches healthy behaviors to the students.

Similarly, dental diseases prevention work is also needed. Pit and fissure sealant for caries prevention is not available and most Tibetans do not know its benefits. Also dental clinics and dentists were few in Tibet, and even fewer in rural areas. When present, the required dental equipments were always inadequate and the dentists, limited by lack of knowledge and modern training, could only provide simple treatment and mostly dental extraction. This may explain the reason we found that many students lost their permanent teeth at a young age.

It is important to emphasize that the surveyed populations in this study were only graduating students from senior high schools, which could not reflect the oral health status of the whole Tibetan people. Therefore, future oral epidemiology investigations should be done in Tibet, especially in the next national investigation.

Finally, proposed suggestions were based on the results above. The first suggestion is to strengthen the prevention of dental diseases. The healthcare personnel should be regularly educated and trained about oral hygiene and health. Their ability and enthusiasm on carrying out the work of oral health care for the Tibetans should be improved. In addition, school based oral health promotion to students should be implemented focusing on skill based learning and attitudes towards health [10] because providing appropriate oral health information from an early age within school education program appears necessary to enhance health literacy and lessen the inequalities in dental health [15]. Thus, Tibetan people and their next generation will be aware of the importance of oral health much earlier. The second suggestion is to improve environment for diagnosis and treatment of dental diseases in medical institutions at all levels in Tibet. More dentists and assistants should be trained to work in plateau area so that more Tibetan people could obtain oral health services. It should be encouraged that more healthcare personnel go to rural areas to provide examination and treatment. With all these efforts the Tibetan people’s oral medical needs could be met in the near future.

Overall, the strengthening of oral health promotion and disease prevention and treatment are urgently needed in Tibet. Although achieving international standards for health care in Tibet may take some time, it is important to press on and move forward.

## Conclusion

Tibetan students had higher prevalence of dental diseases and lower oral health awareness. The related main reasons were geographical environment, dietary habit, students’ attitude to oral health, and lack of oral health promotion. Oral health education and local dentists training should be strengthened and improved so as to get effective prevention of dental diseases.

## Competing interests

There is no financial competing interest on this manuscript.

Financial competing interests

• In the past five years we haven’t received any reimbursements, fees, funding, or salary from an organization that may in any way gain or lose financially from the publication of this manuscript, either now or in the future.

• We didn’t hold any stocks or shares in an organization that may in any way gain or lose financially from the publication of this manuscript, either now or in the future.

• We are not currently applying for any patents relating to the content of the manuscript. We haven’t received any reimbursements, fees, funding, or salary from an organization that holds or has applied for patents relating to the content of the manuscript.

• We do not have any other financial competing interests.

Non-financial competing interests.

• There are not any non-financial competing interests (political, personal, religious, ideological, academic, intellectual, commercial or any other) to declare in relation to this manuscript.

## Authors’ contributions

RH, YM and QX designed study to make an investigation on oral health of Tibetan students. GX, YZ, YW and WY checked the students and made the questionaire investigation. FW, YM and PX collected the data and make the statistical analysis. RH, YM and QX drafted paper. All authors read and approved the final manuscript.

## Authors’ information

Rui Hou and Yong Mi are co-first authors.
